# Avoiding harm in pediatric heatstroke: Lessons from a case of ice-related frostbite

**DOI:** 10.1016/j.jpra.2025.07.009

**Published:** 2025-07-18

**Authors:** Mor Rittblat, Shiran Katabi, Dmitry Kotovich, Stav S. Cahan, Ysh-ia Langer

**Affiliations:** a Israeli Defense Forces Medical Corps, Tel Hashomer, Ramat Gan, Israel; b Department of Plastic and Reconstructive Surgery, Burn and hand units, Hadassah Hebrew University Medical Center, Jerusalem, Israel; c Department of Military Medicine and ``Tzameret,'' Faculty of Medicine, The Hebrew University of Jerusalem, Jerusalem, Israel; d Section of Pediatric Critical Care, Hadassah Hebrew University Medical Center, Jerusalem, Israel

**Keywords:** Frostbite injuries, Heatstroke, Pediatric, Iatrogenic, Cooling, Intensive care

## Abstract

**Background:**

Heatstroke is a life-threatening medical emergency characterized by a core body temperature exceeding 40 °C (104°F). Prompt and effective cooling is critical to prevent multiorgan failure and death. However, aggressive cooling techniques, if improperly applied, may lead to iatrogenic complications. We present a pediatric case of severe frostbite injury resulting from direct ice pad application during emergency treatment for heatstroke.

**Case presentation:**

A 22-month-old infant was found unconscious after being left unattended in a parked car and was diagnosed with severe heatstroke. During prehospital care, direct ice packs were applied per emergency protocol. On admission, erythematous skin lesions were noted on both anterior thighs, which progressed to full-thickness eschars. The plastic surgery team later confirmed these as frostbite injuries. The patient underwent surgical debridement and autologous skin grafting, followed by successful recovery without long-term sequelae.

**Conclusion:**

This case underscores the potential risks associated with rapid cooling interventions, particularly in pediatric patients with more delicate skin. While immediate temperature reduction is vital in treating heatstroke, cooling methods must be carefully selected and monitored. This report highlights the need for pediatric-specific protocols that prioritize both efficacy and safety, and emphasizes the importance of thorough communication between prehospital and in-hospital providers.

## Introduction

Heatstroke is a medical emergency defined by core body temperature 40 °C (104 °F) often due to environmental heat exposure or exertion. It causes systemic inflammatory response, multiorgan dysfunction, and CNS damage.^1^ The pathophysiology of heatstroke involves the failure of thermoregulatory mechanisms,^2^ leading to systemic inflammatory response, cellular injury, and multiorgan dysfunction.^1^ Clinical manifestations include altered mental status, ranging from confusion to coma, hot and dry skin, and potential progression to rhabdomyolysis and disseminated intravascular coagulation.^2^ The pediatric population is prone to rapid heatstroke development due to the immature thermoregulatory systems, higher metabolic rate, inefficient sweating mechanisms, and greater surface area-to-body mass ratio.^3^

Prompt cooling is lifesaving, but method selection is critical. Direct ice application, though historically used, may cause cold-induced injuries, particularly in pediatric patients with delicate skin. We present a case where the initial cooling method used in the field was not clearly communicated, leading to a diagnostic challenge. This report aims to raise awareness of cold-induced injuries as a possible complication of emergency cooling, and to highlight the importance of clear communication and vigilance in early diagnosis.

## Case report

**Figure 1:**
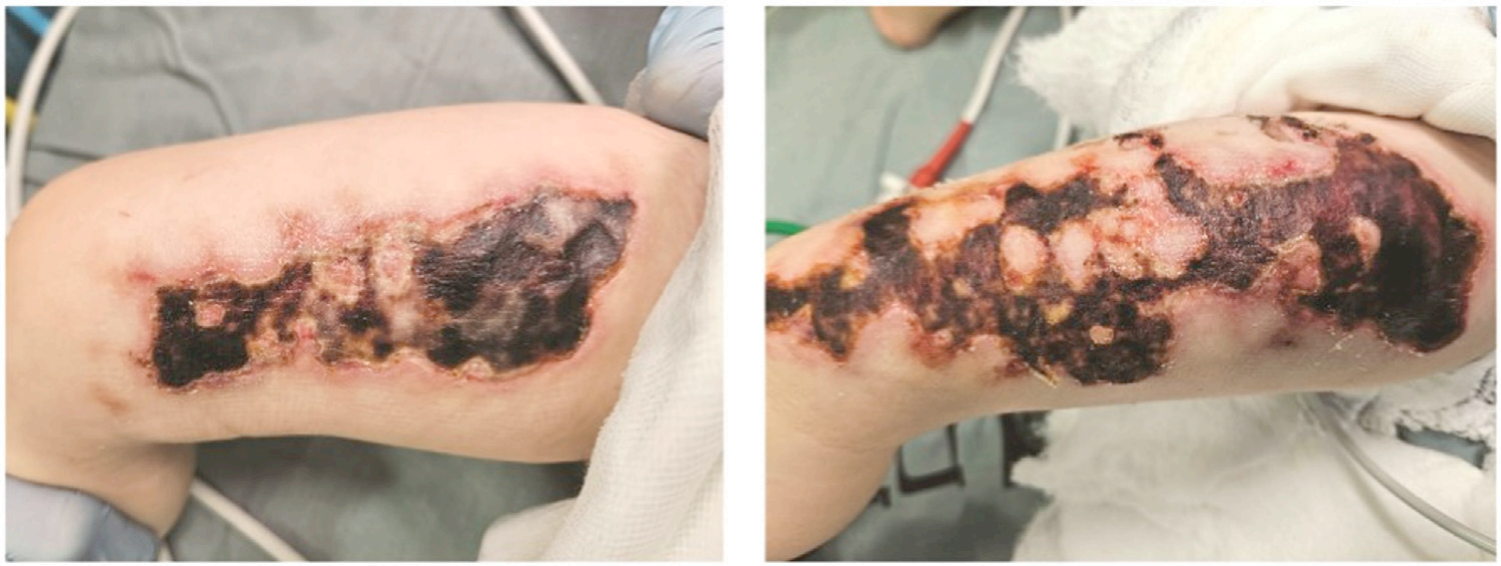
A photo of the preoperative lesions a few days into the Pediatric Intensive Care Unit admission.

A 22-month-old girl presented in our institute after being left unattended in a parked car for several hours. She was rescued and brought to the pediatric emergency department (PED) by Emergency Medical Services (EMS). Upon transport, ice was applied in order to reduce core temperature. On arrival to the emergency department, she exhibited signs of severe heatstroke, including hyperthermia, altered consciousness, tachycardia and hypotension. During stabilization erythema was noticed on both anterior thighs, initially thought to be thermal burns from sun exposure or sun-heated surfaces (e.g., metal buckles), delaying recognition of frostbite. A few days into the Pediatric Intensive Care Unit (PICU) admission, these lesions developed to sever full thickness eschar as shown in Figure 1.

**Figure 2:**
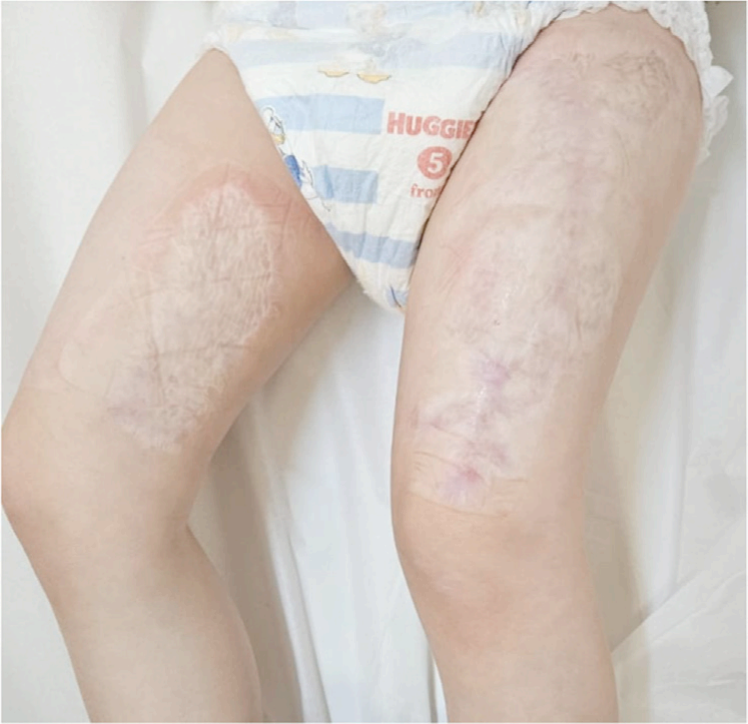
A photo of the follow-up visit one year postoperatively at the burn clinic.

Given the clinical course, including the rapid progression from local erythema to full-thickness eschar, and the application of ice during transport, the diagnosis of frostbite was made by the plastic surgery team in the burn unit. The burns affected 5 % of her total body surface area (TBSA), with 3 % on the left thigh and 2 % on the right thigh. The patient underwent surgical debridement of burn wounds followed by autologous skin grafting on postburn day 15, using split-thickness skin from the right thigh. The surgery was successful (Figure 1S), and the postoperative course was uneventful. The patient was discharged and continued follow-up care at the burn clinic (Figures 2S and 3S). At her follow-up visit at one year postoperatively, the patient demonstrated normal scar healing and achieved full clinical recovery without any long-term complications or wound-related sequelae as shown Figure 2.

## Discussion

Heatstroke is a life-threatening emergency requiring prompt diagnosis and rapid cooling. Pediatric patients are especially vulnerable due to immature thermoregulation, and higher surface area-to-mass ratios.^1^ Rapid reduction of core temperature remains the cornerstone of management, but the method must balance efficacy with safety.

Whole-body immersion in ice water is considered the most effective cooling method in adults, with high cooling rates (0.20–0.35 °C/min).^2^^,^^4^ It’s the preferred method for healthy, young, and well-trained athletes.^2^ However, it poses risks in young children, including cold shock, arrhythmias, and skin injury.^3^^,^^4^ Similarly, ice pack application—especially when unwrapped or applied for prolonged periods—can cause frostbite, as illustrated in our case. However, when used strategically, they should be wrapped in towels and moved frequently.^5^ Additional in-direct, evaporative cooling techniques, which use air-conditioning, fans and misting devices, enhance heat loss from the skin surface by leveraging the high latent heat of vaporization.^4^

Controversy exists over what therapeutic modality is most effective in the treatment of heatstroke. Yet, the basic premise of rapidly lowering the core temperature to 39 °C remains the primary goal.^1^^,^^2^ To date, there is limited knowledge regarding the management of heatstroke in the pediatric population compared to adults.^4^ We recommend: (1) avoiding direct ice application in pediatric patients, (2) prioritizing evaporative cooling techniques, and (3) adopting conservative cooling rates (∼0.10 °C/min). Next steps include developing pediatric-specific EMS protocols, training providers in safe cooling practices, and encouraging structured communication between prehospital and in-hospital teams.

## Conclusion

While direct ice contact is a well-known risk factor for skin injury, this case highlights a more subtle diagnostic and communication challenge. The frostbite was initially unrecognized, with early signs misinterpreted as thermal burns due to prolonged sun exposure or contact with hot metal surfaces. The delay in identifying the true etiology was partly due to incomplete communication regarding prehospital interventions. This underscores the need for heightened clinical suspicion of frostbite during heatstroke treatment, and emphasizes the need for safer, pediatric-specific cooling protocols. We recommend caution with direct-contact cooling and prioritization of evaporative methods, along with caregiver and provider education to enhance safety and outcomes.

## Author contributions

MR—Data curation, Investigation, Writing—original draft; YL—Conceptualization, Data curation, Investigation, Project administration, Supervision, Validation, Visualization, Writing—original draft, Writing—review and editing; SK—Writing—original draft; DK—Writing—original draft; SCC—Conceptualization, Data curation, Investigation, Project administration, Supervision, Validation, Visualization, Writing—review and editing.

## Financial support

The authors report no financial support to declare.

## Data availability statement

Full demographic data pertaining to individuals cannot be disclosed in order to ensure subjects’ anonymity and data security policies. Derived data supporting the findings of this study may be made available from the corresponding author MR upon request.

## Ethics, consent to participate, and consent to publish declarations

Written informed consent for publication of the clinical details and clinical images was obtained from the guardian of the patient.

## Declaration of competing interest

MR, YL, SK, DK, SSC reports no conflict of interest.

## Appendix Supplementary materials

Image, application 1

Image, application 2

Image, application 3
