# Supplementary figures and images for: Avoiding harm in pediatric heatstroke: Lessons from a case of ice-related frostbite
Source: JPRAS Open. 2025 Jul 18;45:386–9. doi: 10.1016/j.jpra.2025.07.009 (PMC12340393; doi:10.1016/j.jpra.2025.07.009)

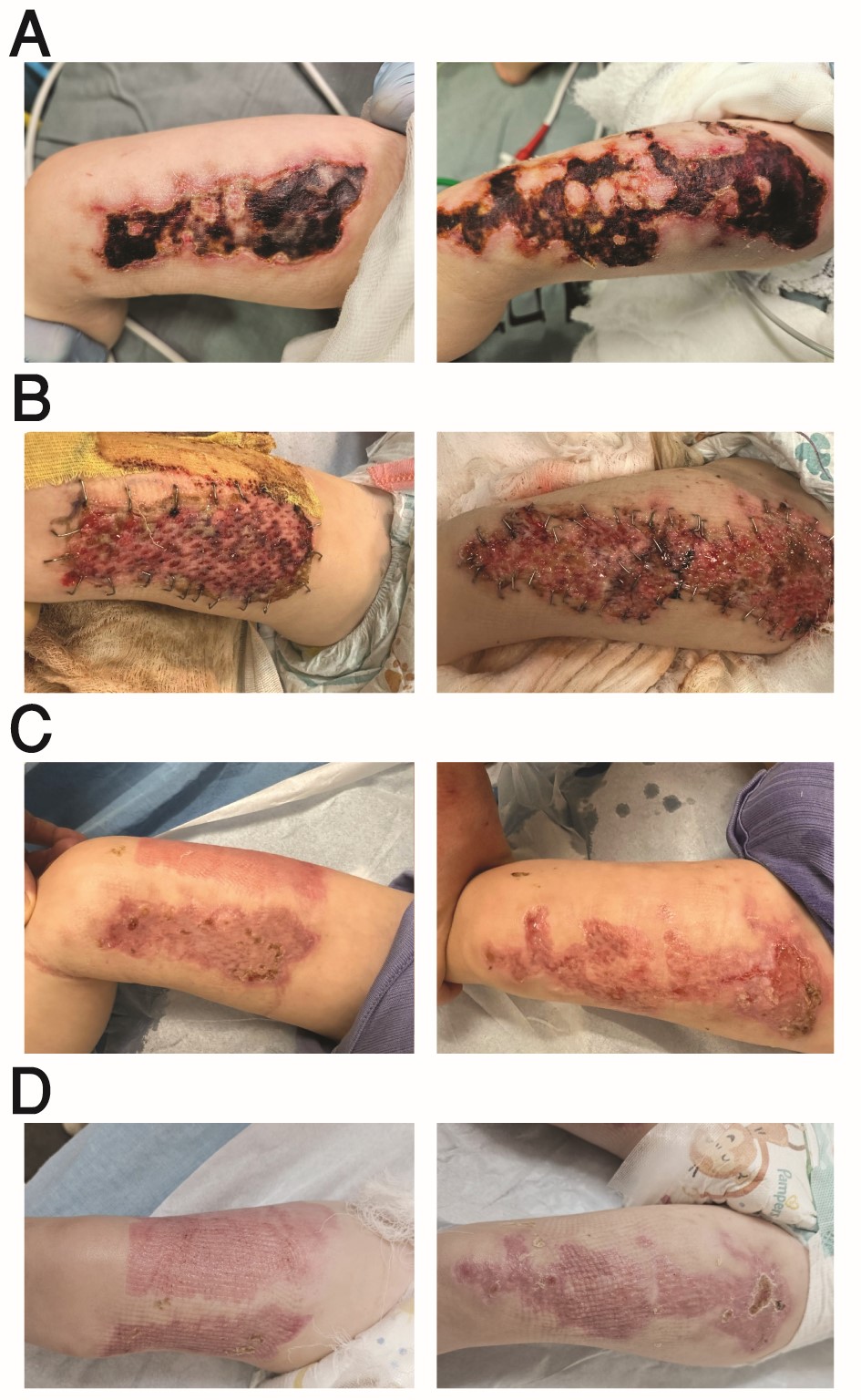


Figure 1S – A photo of the lesions after autologous split-thickness skin grafting

Supplement: Supplementary file 1 [file mmc1.docx]

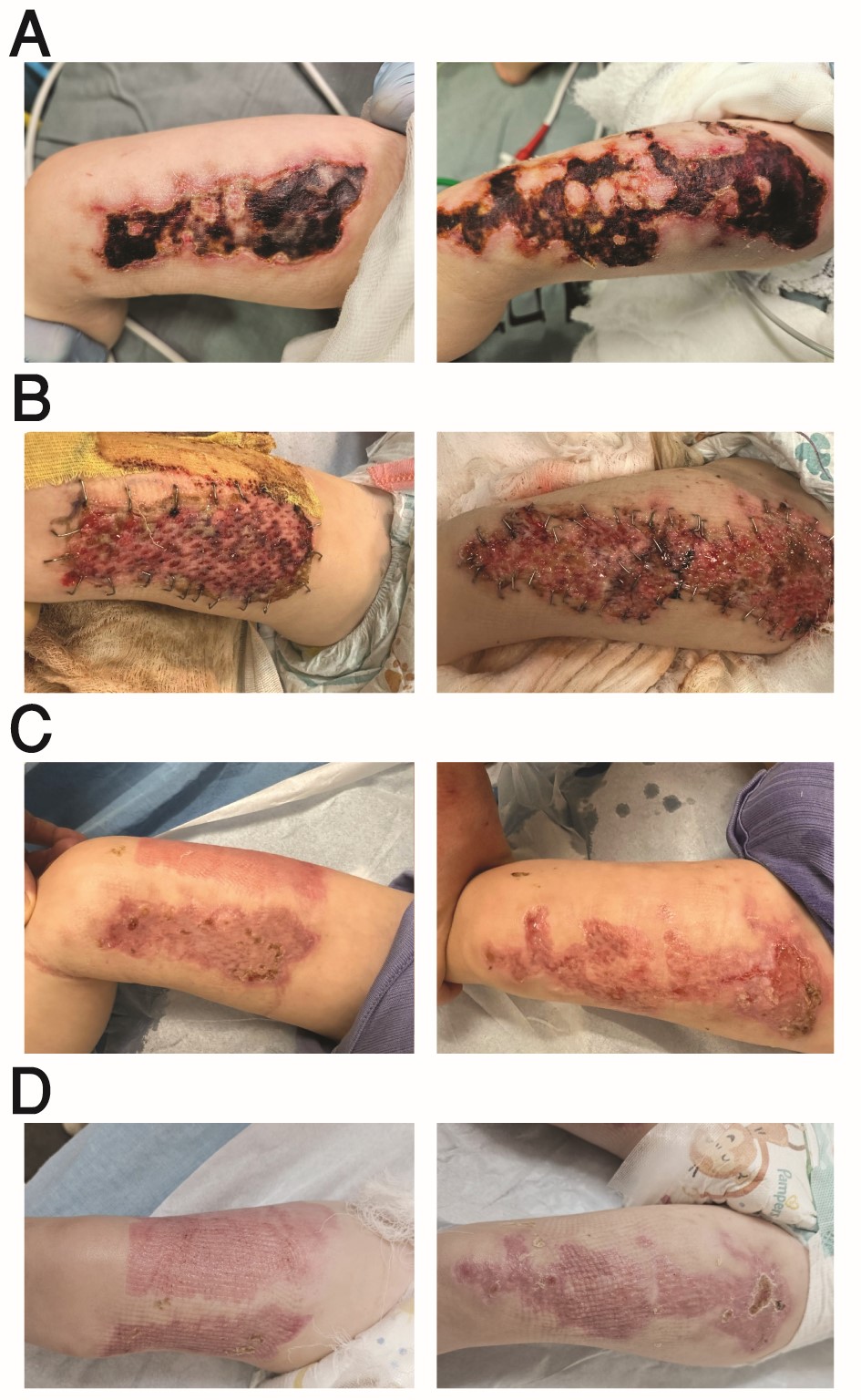


Figure 2S – A photo approximately a month after the follow-up care at the burn clinic

Supplement: Supplementary file 2 [file mmc2.docx]

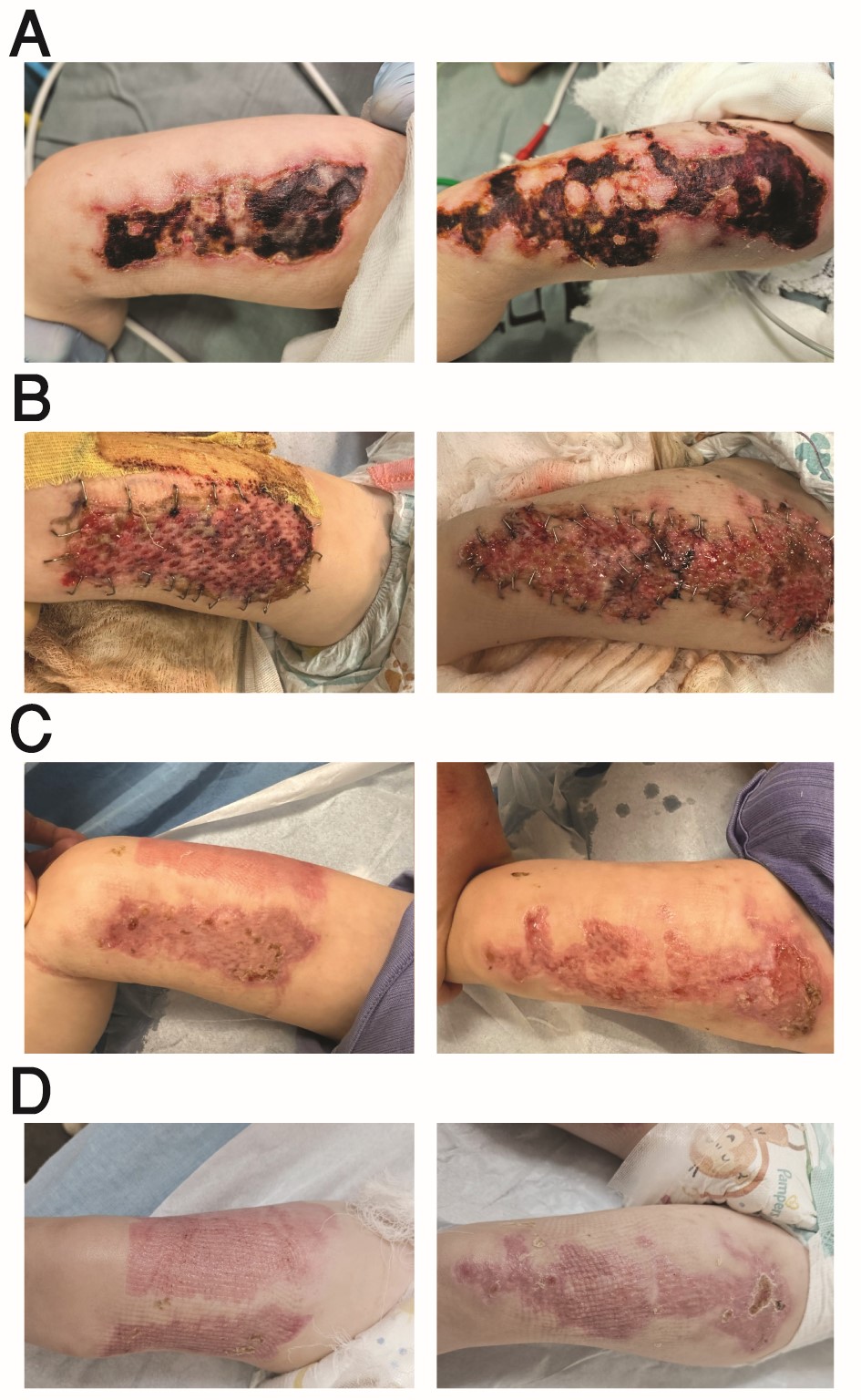


Figure 3S - A photo approximately three months after the follow-up care at the burn clinic

Supplement: Supplementary file 3 [file mmc3.docx]
